# Atrial fibrillation source area probability mapping using electrogram patterns of multipole catheters

**DOI:** 10.1186/s12938-020-00769-0

**Published:** 2020-05-05

**Authors:** Prasanth Ganesan, Elizabeth M. Cherry, David T. Huang, Arkady M. Pertsov, Behnaz Ghoraani

**Affiliations:** 1 Department of Computer and Electrical Engineering, Florida Atlantic University, Boca Raton, FL USA; 2 School of Computational Science and Engineering, Georgia Institute of Technology, Atlanta, GA USA; 3 Department of Cardiology, University of Rochester Medical Center, Rochester, NY USA; 4 Department of Pharmacology, SUNY Upstate Medical Center, Syracuse, NY USA

**Keywords:** Atrial fibrillation, Electrogram signal analysis, Patient-specific therapy

## Abstract

**Background:**

Catheter ablation therapy involving isolation of pulmonary veins (PVs) from the left atrium is performed to terminate atrial fibrillation (AF). Unfortunately, standalone PV isolation procedure has shown to be a suboptimal success with AF continuation or recurrence. One reason, especially in patients with persistent or high-burden paroxysmal AF, is known to be due to the formation of repeating-pattern AF sources with a meandering core inside the atria. However, there is a need for accurate mapping and localization of these sources during catheter ablation.

**Methods:**

A novel AF source area probability (ASAP) mapping algorithm was developed and evaluated in 2D and 3D atrial simulated tissues with various arrhythmia scenarios and a retrospective study with three cases of clinical human AF. The ASAP mapping analyzes the electrograms collected from a multipole diagnostic catheter that is commonly used during catheter ablation procedure to intelligently sample the atria and delineate the trajectory path of a meandering repeating-pattern AF source. ASAP starts by placing the diagnostic catheter at an arbitrary location in the atria. It analyzes the recorded bipolar electrograms to build an ASAP map over the atrium anatomy and suggests an optimal location for the subsequent catheter location. ASAP then determines from the constructed ASAP map if an AF source has been delineated. If so, the catheter navigation is stopped and the algorithm provides the area of the AF source. Otherwise, the catheter is navigated to the suggested location, and the process is continued until an AF-source area is delineated.

**Results:**

ASAP delineated the AF source in over 95% of the simulated human AF cases within less than eight catheter placements regardless of the initial catheter placement. The success of ASAP in the clinical AF was confirmed by the ablation outcomes and the electrogram patterns at the delineated area.

**Conclusion:**

Our analysis indicates the potential of the ASAP mapping to provide accurate information about the area of the meandering repeating-pattern AF sources as AF ablation targets for effective AF termination. Our algorithm could improve the success of AF catheter ablation therapy by locating and subsequently targeting patient-specific and repeating-pattern AF sources inside the atria.

## Background

Atrial fibrillation (AF) is a commonly occurring heart rhythm disorder affecting millions of people every year in the United States as well as worldwide [1]. For several decades, patients with drug-refractory persistent or high-burden paroxysmal AF have been treated using catheter ablation therapy. In this procedure, a multi-pole mapping catheter is maneuvered across the atrial endocardium to create an electroanatomic map of the atrium. Pulmonary vein (PV) isolation procedure is performed to block the abnormal triggers originating from the PVs, typically using the generated map [2, 3]. However, standalone PV isolation procedure has demonstrated only suboptimal success rates [4, 5].

It has been evident from various human and animal studies that repeating-pattern AF sources such as reentry sources with meandering core and focal sources exist in the atrial wall during many cases of AF [6–9]. Ablating these sources, in conjunction with PV isolation, could potentially lead to a higher long-term success rate of catheter ablation [10, 11]. An existing technology to map these sources is the focal impulse and rotor modulation known as FIRM mapping (Abbott Laboratories, IL) [6]. In this mapping technique, a whole-atrial 64-pole, contact basket catheter is deployed in the atrium. The 64-unipolar signals are recorded and processed using a proprietary algorithm based on phase mapping, which then reveals a rotor or a focal source if it is present. However, this technology is primarily dependent on whole-chamber, contact basket catheters with inherent shortcomings, such as low spatial resolution, incomplete atrial coverage, and poor electrode contact, which could be the reason for contradictory success rates at different centers [12]. Another approach, CartoFinder (Biosense Webster Inc., CA), uses either a 64-unipole basket catheter or a 20-unipole diagnostic catheter’s signals to locate reentries and focal impulses based on a combination of wavelet and phase analysis algorithm. However, this approach increases the procedure time by requiring the laborious process of point-by-point coverage of the entire atrial chamber as well as recording 30 s of electrograms at each placement. Other technologies use mathematical cardiac modeling to locate AF sources. In AcQMap (Acutus Medical Inc., CA), the system locates AF sources from dipole densities computed using the biopotential and ultrasound signals collected from a non-contact basket catheter. Another technology is the CardioInsight mapping system (Medtronic PLC., MN), which locates AF sources according to the inverse solution of the epicardium atrial potentials on the patient’s atrial computerized tomography images using 252 surface electrocardiogram signals that are collected using an electrode vest. Although the method has potential for detecting AF sources, the lack of electroanatomic mapping in this technique is still raising concerns about ablation accuracy assessment, low-voltage signal capturing, and inverse-solution accuracy in the presence of fibrosis [13, 14].

In addition to these technologies, a significant amount of research has been performed on the design of computational AF mapping systems that can help to locate AF sources. These methods map the entire area using a single or multiple pole diagnostic catheter and then use some electrogram characteristics such as complex fractionation [15], dominant frequency [16], voltage [17], or local activations delays [18, 19] to detect potential AF sources. In previous work, we developed a novel mapping method for repeating‐pattern AF source localization that does not involve electrophysiological mapping of the entire atria. It gradually navigates a 20-electrode circular catheter towards a repeating-pattern AF source and stops when the center of the catheter is placed within 4 mm of the core of an AF reentry or focal source [20, 21]. However, this method locates only one point on the trajectory path of the source, which could be limiting its applicability to locating meandering AF sources that have been commonly evidenced in human AF [9]. Moreover, the method navigates the catheter center by catheter radius, which may result in several catheter placements and extend the source detection time if the initial catheter placement is far from a source.

In this paper, we describe a mapping technique referred to as the AF source area probability (ASAP) mapping algorithm, which is fundamentally different from the existing approaches in three main aspects: (1) ASAP uses variations in electrogram characteristics at every catheter placement to intelligently sample the atria within a few catheter placements, instead of mapping the entire atria as performed in [15–19] or moving the catheter by one catheter radius as done in [20, 21]; (2) it delineates the trajectory path of the core of a meandering AF source instead of locating one point on the path as performed in previous methods; and (3) it provides a spatial probability map of the presence of the core of a meandering AF source as the catheter is placed in the atria. These novel aspects of the ASAP mapping have the potential to improve clinical human AF ablation by delineating the area of meandering repeating-pattern AF sources within a few catheter placements instead of mapping the entire atria, which is time-consuming and often requires the acquisition of thousands of local electrograms. ASAP provides a visual feedback to the clinician about the probability of the presence of a meandering AF source by overlaying the constructed ASAP map on the atrium anatomy as the clinician maneuvers the catheter inside the atria. ASAP uses an algorithmic approach similar to what has been used in robotic search and motion-planning algorithms that are based on some heuristics to guide the catheter within an optimal number of iterations independent of how far the catheter is from the AF source [22]. We demonstrate that the collective analysis of the information from multiple catheter placements as guided by the electrogram patterns can locate the meandering area of an AF source in the atria regardless of the source type (i.e., reentry or focal) and wave-break patterns even in cases where the existing FIRM mapping technology is challenged. The proof-of-concept feasibility of our algorithm was also investigated using three retrospective cases of clinical AF.

## Results

The ASAP algorithm guides the sequential movements of a 20-pole diagnostic catheter from an arbitrary initial placement on the atrial tissue until an area with a high probability of the presence of the meandering core of an AF reentry or focal source is detected or a maximum of 12 catheter placements has occurred. At every catheter placement, *k*, ASAP analyzes the patterns of the recorded electrograms to estimate the principal wave direction (PWD), as the direction opposite to the direction of the propagating AF source wavefront relative to the current location of the catheter (Phase I), and accordingly updates an ASAP map on the atrial anatomy (Phase II). The ASAP map provides the spatial distribution of the probability of the meandering core of an AF source on the tissue. Based on a set of source detection criteria on the updated map, the algorithm determines whether the trajectory path of a meandering AF source is delineated or the search has to be continued (Phase III). In the former case, ASAP stops the search and provides the area of the trajectory path of the meandering AF source $A_{H}^{\left( k \right)}$, and in the latter scenario, it provides a recommendation for the location of the next catheter placement (Phase IV).

**Fig. 1:**
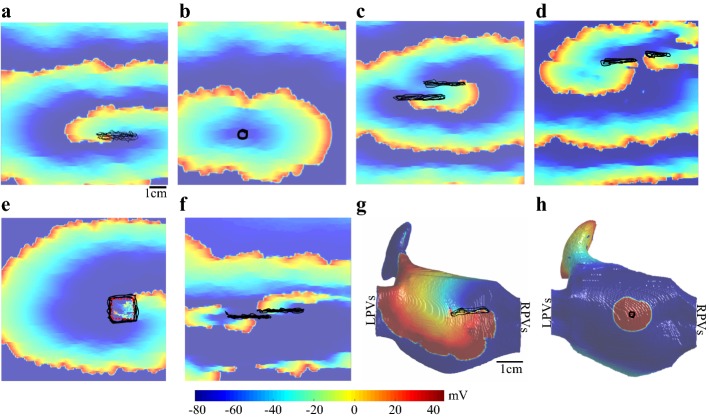
Human AF simulations. **a** Single reentry source with cycle length (CL) ≈ 140 ms. **b** Single focal source with CL ≈ 150 ms. **c** Figure-of-eight reentry with CL ≈ 180 ms. **d** Reentry source in two-layer model with CL ≈ 140 ms. **e** Rotor source anchored to a 2.25-cm^2^ myocardial patchy scar (indicated by a red dashed box), CL ≈ 245 ms. **f** Stable fibrillatory cluster of rotors, CL ≈ 170 ms. **g** Single reentry source in 3D real anatomy with CL ≈ 170 ms. **h** Single focal source in 3D real anatomy with CL ≈ 250 ms. The black tracings indicate the tip of the meandering source trajectory with longer lifespans. The colors represent the transmembrane voltage by the color scale in mV. *LPV* left pulmonary vein, *RPV* right pulmonary vein

The method was evaluated on a set of 2D and 3D human AF simulation cases with different arrhythmia scenarios (shown in Fig. 1) and three retrospective clinical AF cases. Additional file 1: Movie S1, Additional file 2: Movie S2, and Additional file 3: Movie S3 demonstrate the complexity and wave collision in three simulations. A few samples of fractionated electrograms generated and used in this study are shown in Additional file 4: Figure S1. The ASAP algorithm was implemented in MATLAB R2017b and the AF simulations were implemented in Fortran. The algorithm and 2D simulations were performed on Dell precision workstation T5810XL with Intel Xeon Processor E5-1620 v3 (3.5 GHz) and 64 GB RAM, and simulating the 3D cases were executed on comet at the San Diego Supercomputing Center. Please refer to the comet website [23] for a description of the technical specs.

**Fig. 2:**
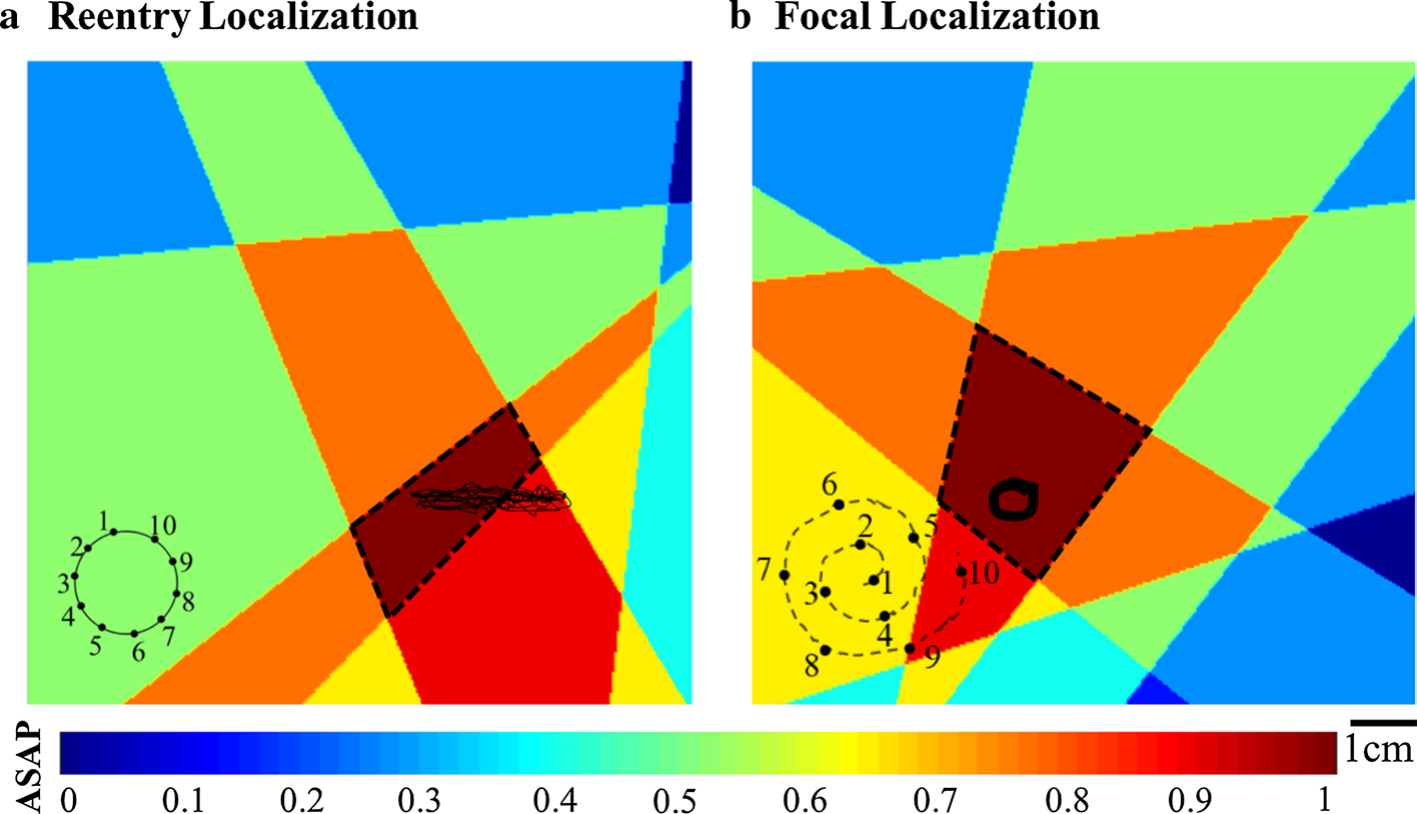
Examples of reentry and focal source localization. **a** Final ASAP map obtained using a Lasso catheter with a reentry source (simulation case of Fig. 1a). **b** Final AF source area probability (ASAP) map obtained using a spiral catheter with a focal source (simulation case of Fig. 1b). The black dotted boundary indicates the high-probability (HP) area in both cases. The black tracings indicate the trajectory of the source core

The ASAP algorithm provided $A_{H}^{\left( k \right)}$, the boundary coordinates of the highest probability ASAP map as the delineated area of a meandering AF source. The algorithm was considered successful if at least 4 mm (an ablation catheter tip diameter) of the trajectory of the meandering reentry source was inside the detected area $A_{H}^{\left( k \right)}$. In case of a focal AF source, the algorithm was considered successful if the detected area included the core of the source. Otherwise, the algorithm was considered a false detection. A detection failure was when the algorithm did not delineate any AF source within $k$ = 12 catheter placements or the delineated area in 1.5 ${\text{Th}}_{1}$ (i.e., three times of the catheter area). A case of AF reentry source localization (simulation case of Fig. 1a) using a Lasso catheter is shown in Fig. 2a, and an AF focal source location (simulation case of Fig. 1b) using a spiral catheter is illustrated in Fig. 2b. Both cases are considered a successful delineation because the ASAP source detected area included 1.5 cm of the reentry trajectory and the core of the focal source. We tested the algorithm performance from different initial catheter placements. The average successful detection rates for all the possible initial catheter locations across the atrial tissue were calculated for the reentry and focal sources (i.e., simulations in Fig. 1a, b, respectively) and are provided in Table 1. The results are provided using both Lasso and spiral catheters. The average successful detection was greater than 95% for all experiments, which means that ASAP successfully located the AF source in the majority of the cases. The algorithm detected a source area at every trial, so it did not result in any detection failure. In case of an AF reentry, we also calculated the length of the trajectory path of the source core that was inside the detected area to investigate the algorithm’s precision in delineating a meandering AF source. As reported in Table 1, the normalized length of the trajectory of the reentry source inside the detected area was 0.87 ± 0.09 mm when using the Lasso catheter and 0.80 ± 0.12 mm for the spiral catheter. Note that all the delineated ASAP areas included at least some portion of the reentry trajectory, although some areas included less than 4 mm of the trajectory or the delineated area was greater than three times of the catheter area and thus were classified as a false detection.

**Table 1:** Summary of performance of the algorithm for various simulation cases

Simulation type	Catheter type	Successful delineation (%)	Normalized length of trajectory in delineated AF area (mean length)	Difference in PWD and true direction
Rotor source	Lasso	99.98	0.87 ± 0.09 (21.8 mm)	23.76° ± 17.75°
	Spiral	99.99	0.80 ± 0.12 (20 mm)	33.80° ± 17.85°
Focal source	Lasso	100	N/A	25.17° ± 15.64°
	Spiral	100	N/A	34.00° ± 15.77°
Figure of eight	Lasso	97.01	0.66 ± 0.03 (23.1 mm)	23.93° ± 12.41°
Two layer model	Lasso	95.65	0.64 ± 0.26 (12.8 mm)	25.50° ± 19.10°
Conduction block	Lasso	98.57	0.83 ± 0.11 (45.9 mm)	24.54° ± 21.73°
Fibrillatory rotor cluster	Lasso	96.02	0.71 ± 0.29 (20.7 mm)	24.47° ± 20.30°
Rotor source (3D)	Lasso	98.10	0.73 ± 0.10 (10.9 mm)	26.26° ± 19.43°
Focal source (3D)	Lasso	99.41	N/A	25.02° ± 12.04°

The fourth column shows the normalized length of the trajectory of the meandering AF source present inside the highest probability area; for example, a value of 0.87 implies that 87% of the reentry trajectory was present inside the area and the 87% length value is 21.8 mm. Both normalized length and the actual length are averaged over all the algorithm runs. The last column shows the difference between the calculated principal wave direction (PWD) angle and the actual angle in degrees—for reference, the angle between a bipole of a Lasso is about 35° and a spiral has an outer bipole angle of about 56°

**Fig. 3:**
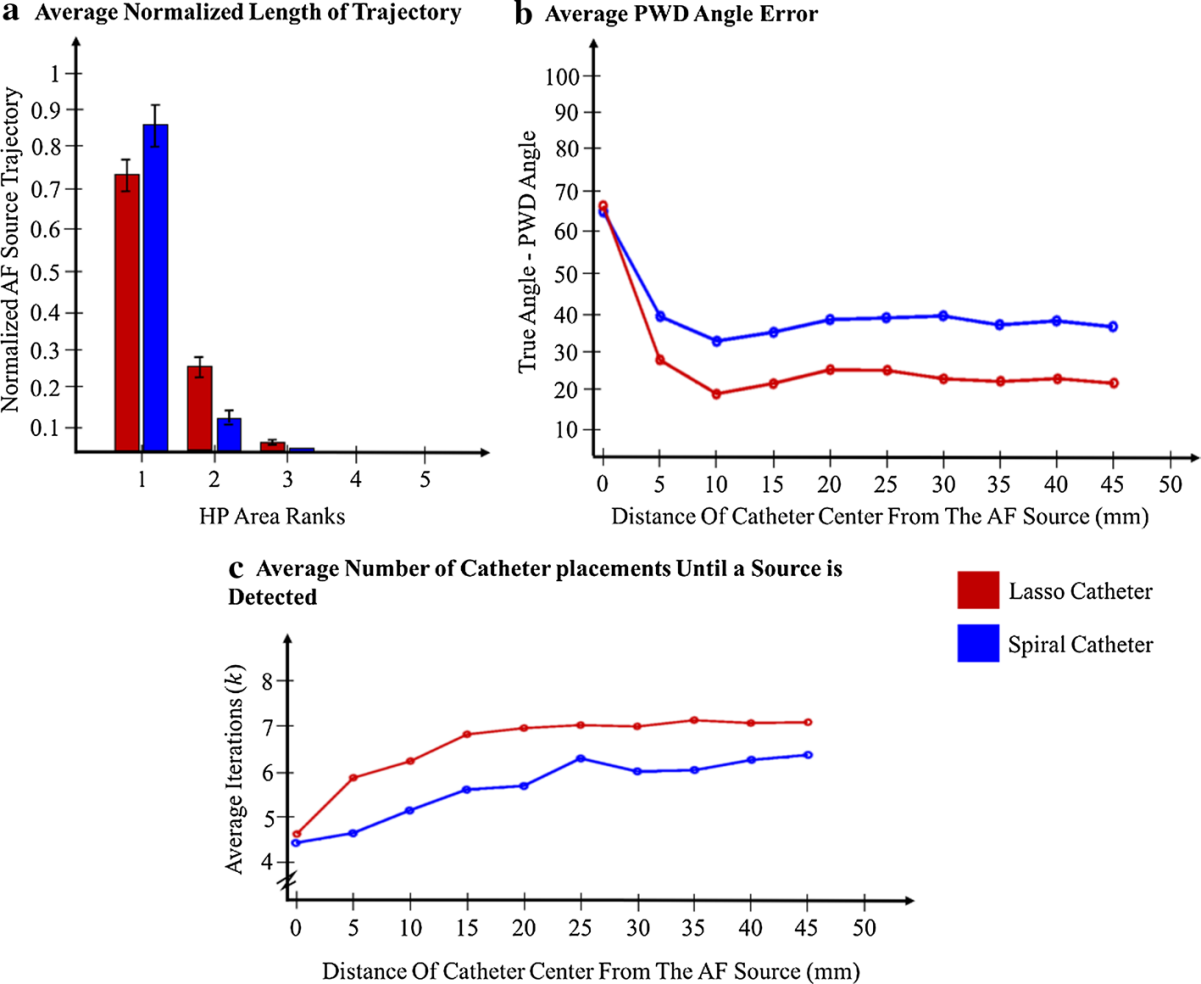
**a** Each bar represents a normalized trajectory length present in each probability area. **b** Difference between the calculated principal wave direction (PWD) angle and the true angle of the AF source. **c** Average iterations until a source detection. The values were averaged over focal and reentry cases (simulation cases of Fig. 1a, b). *HP* high probability

Next, we investigated the distribution of the AF source trajectory in different regions of the ASAP map. For this purpose, we sort the probability values of the final ASAP map from the largest to the smallest value and plot the length of the trajectory of the source that exists in each probability value. The results are provided in Fig. 3a. Note that the majority of the AF source trajectories were located in the first or second highest probability ASAP map area, and less than 5% of the source trajectory was present in the third highest area. No trajectory was present in the other ASAP areas.

In our next analysis, we investigated the efficacy of the algorithm in detecting the wave propagation direction. For this purpose, we calculated the difference between the angles of the calculated PWD and the actual PWD (i.e., a vector starting from the catheter center to the source trajectory center) over the distance of the catheter center from the source trajectory center. A small difference between the two PWD vectors’ angles indicates that the algorithm was effective in estimating the direction of the wave propagation. The results are shown in Fig. 3b. The PWD angles of the Lasso catheter was always < 30° for distances ≥ 4 mm from the source, and for distances < 4 mm the PWD angles are < 70°. Note that the algorithm relies on the estimated PWD when the catheter is far from the AF source, so the larger PWD estimation error does not affect ASAP. In the case of the spiral catheter, the angle values are higher than for the Lasso due to larger inter-electrode spacing.

Our next step in the analysis is to investigate the efficiency of the algorithm in delineating the meandering area of an AF source. For this purpose, the average number of catheter placements until a source was detected is shown in Fig. 3c. An interesting observation is that the algorithm guided the catheter using fewer than eight catheter placements independent of the initial catheter’s location with respect to the AF source. Such a small number of catheter placements indicates the algorithm’s speed and efficiency in detecting AF sources.

**Fig. 4:**
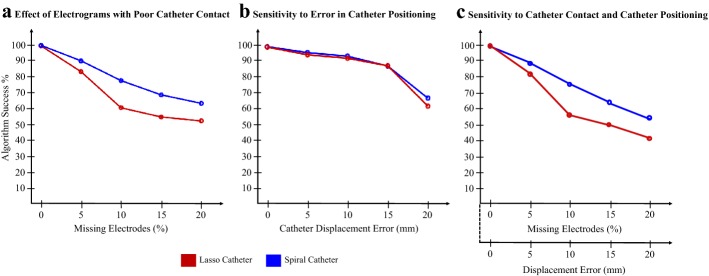
**a** Electrograms were randomly made isoelectric to simulate the scenario of a poor catheter contact. As the percentage of the missing electrodes increases, the algorithm success gradually decreases. **b** Catheter-placement error was introduced in the algorithm-recommended location for navigation. **c** The effect of simultaneous errors due to poor contact of electrodes and catheter displacement in the AF detection accuracy of the algorithm. The values were averaged over focal and reentry cases (simulation cases of Fig. 1a, b)

We validated the practicality of our algorithm by investigating its sensitivity to poor electrode contact and displacement of the catheter from the algorithm-recommended placement. The effect of missing electrodes due to poor catheter contact was simulated by randomly removing one or two electrodes from the algorithm. The change in the successful detection with respect to the percentage of the missing electrodes is shown in Fig. 4a. As expected, the successful delineation was found to decrease as the percentage of missing electrodes increases. Note that the spiral catheter was more robust to missing electrodes compared to the Lasso catheter, possibly because when the electrode was missing in one of the loops of the spiral catheter, the PWD angle was still accurately estimated by the electrode in the other loop. Next, the catheter misplacement error was simulated by placing the catheter at $P^{{\left( {k + 1} \right)}} + \left( {\varepsilon_{x} ,\varepsilon_{y} } \right)$, where $\varepsilon$ is selected randomly from a uniform distribution $\in \left[ {0,r} \right]$. Figure 4b provides the change in the rate of successful detection as $r$ increases from 0 (no catheter positioning error) to 20 mm. The results show that the success rate of the algorithm does not go below 85% even for 15 mm placement error for both catheter types. Next, we investigated the effect of both poor contact and catheter-positioning error on the method’s performance. The result, shown in Fig. 4c, demonstrates a similar behavior meaning that the success rate declines with an increase in the catheter-placement error and the missing electrode rate. However, application of a spiral catheter is more promising than a Lasso catheter. With the spiral catheter, ASAP achieved a 90% success rate with the 5% missing electrodes and 5 mm displacement from the recommended catheter placement.

We further investigated the algorithm by testing on various reentry‐driven atrial arrhythmia models: figure-of-eight (Fig. 1c), two-layer (Fig. 1d), macroreentry reentry anchored to an anatomical barrier (Fig. 1e), and stable fibrillary cluster of reentry activities (Fig. 1f). The detection success rates are reported in Table 1. The success rates were greater than 95% even for these complex AF cases.

In addition to the 2D simulations, we validated ASAP on 3D simulation cases. For computational purposes, we applied the ASAP algorithm in a 2D equivalent of the 3D real anatomy. Instead of guiding the catheter in a 3D anatomy, we performed the algorithm computations in its 2D equivalent and transformed the generated ASAP map to the 3D anatomy at every step of the algorithm. Additional file 4: Figure S2 shows this transformation and Additional file 4: Figure S3 shows an example of a step-by-step mapping of a reentry source on the 3D case. The figure shows how the 3D simulated atrium was transformed to a 2D surface and the algorithm was executed in 2D, and then the results at every step were transformed back to the 3D anatomy. The average results for 3D focal and reentry sources are reported in Table 1. As shown in the table, there was no significant drop in the performance of the algorithm in comparison to the 2D cases. This observation indicates the adaptability of the algorithm across different domains of 2D and 3D, which is a major benefit while applying to clinical data.

Next, we compared the performance of ASAP to the performance of the FIRM mapping, an existing clinical method that uses unipolar electrograms that are collected using a basket catheter to map AF sources in the atria. Our comparison was carried out by applying FIRM to four 2D and two 3D simulation cases in Fig. 1. A total of 120 different orientations of a 64-electrode basket catheter (Abbott Electrophysiology) with diameter 40 mm (the highest resolution) were placed on every 2D simulated tissue and three different orientations of the basket catheter were placed in every 3D simulated atrium. The average source detection percentage for each simulation was calculated to be 44.52% (simulation in Fig. 1a), 38.93% (Fig. 1b), 29.46 (Fig. 1c), 54.01% (Fig. 1f), 66.27% (Fig. 1g), and 63.74% in simulation of Fig. 1h. Such a low performance of the FIRM mapping technique is consistent with the observations from the previous studies, which reported a high sensitivity of the method to the catheter resolution and catheter-placement orientation with respect to the AF source or fibrosis orientation [21, 24]. Comparing these detection accuracies with the ASAP mapping performance (Table 1) indicates the potential of ASAP in the successful detection of the AF sources where existing clinical methods fail.

**Fig. 5:**
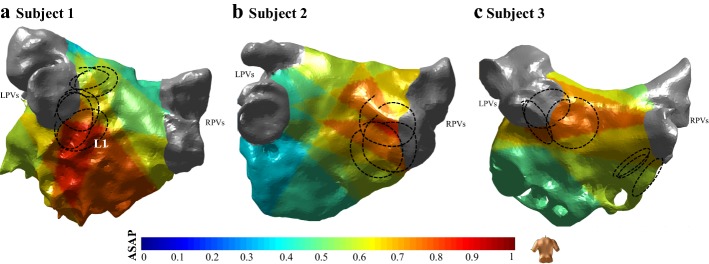
AF source area probability (ASAP) mapping constructed for three subjects. The figures show the posterior view of the left atrium, and the black circles indicate the possible location of reentries as manually labeled by an electrophysiologist according to the criteria in [25]. LPVs: left pulmonary veins. RPVs: right pulmonary veins. The electrogram recordings at catheter placement L1 is shown in Additional file 4: Figure S4

Finally, we also explored the potential of the algorithm for clinical AF cases. Figure 5 shows the ASAP results of the retrospective mapping for 3 AF cases. In each case, an ASAP map was constructed using the existing catheter placements. Given the retrospective nature of our analysis in the case of clinical AF data, we compromised the catheter navigation of the algorithm in Phase IV by moving the catheter to the coordinates of the catheter placement that is closest to the suggested coordinates from the algorithm. The displayed catheters (black dashed circles) in Fig. 5 indicate the possible location of a meandering AF reentry source as defined as sequential temporal local activation around the circular catheter and termination of AF while ablating the area of the AF reentry sources [25]. Several interesting observations can be made. First, by comparing the location of the constructed ASAP maps and the location of a possible AF reentry, we can infer that the highest probability area of the ASAP map overlaps with the location of the possible AF reentry sources. Additional file 4: Figure S4 displays the recording at one of the catheter placements in the delineated area for each subject. A sequential temporal activation pattern around the circular catheter at the delineated area indicates the presence of an AF source. Another interesting observation is that in case of subject 3, ASAP delineated only one of the AF sources (possibly the dominant one during the analysis interval). In prospective clinical studies, the other source would be delineated once the first dominant AF source is ablated. This process is also performed in the FIRM mapping where, first, the dominant source is detected and ablated and then the next dominant source is mapped [6]. Further investigation of the delineated AF sources in these three subjects indicates that all the detected HP areas were around the PVs. The reason is that these three patients underwent their first ablation and the mapping was performed before the ablation was performed. Hence, as expected, the dominant source was in the PV areas and was detected by ASAP. We also studied the coincidence of the ablation sites with the detected AF sources. For all the three subjects, the ablation of the detected AF sources was performed at the time of AF ablation. However, out of these subjects, AF was terminated in the case of subjects 2 and 3, while subject 1 remained in AF after the PV ablation. The ASAP map of subject 1 as shown in Fig. 5a presents a probability of the presence of an AF source in the posterior wall under the inferior left PV, which may indicate the presence of additional AF sources outside the PV areas and the continuation of AF even after PV ablation. However, further investigation requires prospective and real-time application of ASAP in clinical AF ablation procedure after PV ablation.

## Discussion

A novel mapping algorithm was developed to determine the area of reentry or focal sources with high certainty using two types of conventionally used diagnostic catheters. Given that the anatomy map of a patient is provided, ASAP not only builds the probability map of an AF source, but also navigates the catheter to algorithmically calculated locations until it locates the meandering AF source area. The evaluation of the algorithm covered a range of test cases to show the robustness of the algorithm to adapt to complex source patterns with fibrosis in 2D and 3D simulated domains and real clinical domain.

An important factor that determines the accuracy of the algorithm is the reliability of the PWD vector in giving the direction of the source. Since the PWD is an approximate estimated value, it may introduce errors in both catheter navigation (see Eq. (5)) and mapping (see Eq. (2)). From Table 1, it can be seen that the difference between the PWD angle and the true angle of the source has an average error of no greater than the angle between two bipole electrodes. This observation indicates that our PWD estimation method provides a reliable approximation of the source direction. The higher values of the PWD difference when using a spiral catheter are due to the catheter geometry, which has a higher inter-electrode spacing than a Lasso catheter. Additionally, we observed a consistent (almost flat) PWD estimation error when the catheter is placed at any distance greater than 4 mm from the source (Fig. 3b). This observation further demonstrates the robustness of PWD as a measurement of the direction of an AF source. Another important observation from Fig. 3b is that a threshold of $\frac{\pi }{2}$ rad that was used in the mapping step (Phase II of the “Methods” section) is indeed a reasonable selection since all the PWD angles greater than 4 mm from the source are less than 90°.

The robustness of the algorithm also depends on the number of iterations taken to detect an AF source. Any redundant iteration reflects poorly in the clinical scenario by increasing the total procedure time. ASAP takes an average iteration of only 6–7 catheter placements to localize an AF source, with fewer placements needed when the initial distance of the catheter from the source was small (Fig. 3c). The spiral catheter was found to take fewer iterations than the Lasso due to the mapping stop condition, which is based on the area of the catheter (Phase III of the “Methods” section), because the area of the spiral catheter is larger than that of the Lasso.

We also validated ASAP on scenarios with complex wavefronts and atrial geometries. We did not observe a significant decrease in the algorithm’s performance when it was applied to a case of the 3D realistic atrium in both focal and reentry source (Table 1). Such behavior was expected as the wave propagation on the endocardial surface of the 3D simulation is a 2D wave propagation, and the geometry of the 3D atrium does not alter the electrogram patterns on the 2D surface. Hence, as along as ASAP can deal with the complex wave patterns in a 2D surface, it is expected to perform equally well in a 3D atrium. It is also important to note that the ASAP algorithm’s ability to detect both reentry and focal sources indicates that it is also able to detect 3D scroll waves. Note that 3D scroll waves can be divided into two distinct groups, those whose filaments intersect the myocardial surface and those that do not intersect. In case of the former, the scroll wave filament manifests itself as a reentry (Fig. 1a), and in case of the latter, it will appear as a focal activation pattern (Fig. 1b) or a breakthrough (Fig. 1d). Our simulation cases with two layers of myocardium (Fig. 1d) and complex fibrillatory reentry (Fig. 1f) show a chaotic wave breakthrough resulting in two intermittently occurring meandering reentry sources. The simulation case with an anatomical barrier (Fig. 1e) investigates the method’s performance in the presence of scars or low-voltage areas, which are common for pathological atrial myocardium. The validation of the ASAP mapping on these scenarios shows a successful performance of greater than 95%, which suggests that the algorithm can determine the area of meandering AF sources in scenarios of myocardial scars and high transmural fibrosis observed in persistent and long-standing persistent patients [26].

In a clinical setting, it may not be possible to place the catheter with all the electrodes in complete contact with the endocardium. The electrodes with poor contact are generally removed from the analysis as they are contaminated by noise and far-field effect and do not represent the local activations. ASAP was found to be sensitive to catheter contact as the success rate goes below 70% when more than 5% (of the tissue) of electrograms are missing (Fig. 4a). When the percentage of missing electrodes increases, the percentage of PWD error also increases, thereby shifting the high-probability ASAP area to a different location than the actual one. Another practical challenge is that the catheter may not be placed precisely at the location suggested by the algorithm due to the limited steering capability of intra-cardiac catheters. Our analysis showed that the algorithm is robust to errors in catheter placement. The performance of the algorithm varied only ± 10% from the actual success rate as the catheter displacement error was increased (Fig. 4b). We believe that this robustness was achieved mainly because the algorithm is dependent only on PWD and not on the catheter-placement location. However, at very high displacement error, the algorithm’s performance seems to deteriorate. The robustness of the algorithm to both errors in catheter placement and missing electrodes (Fig. 4c) showed a similar behavior in a reduction in the performance with a higher number of electrode with poor contact. However, in general, Lasso and spiral catheters are less likely to exhibit poor contact compared to a spherical basket catheter placed in a non-spherical atrium. Moreover, the current technologies present in Lasso and spiral catheters for contact-force sensing helps to avoid such poor-contact scenarios [27]. The algorithm’s sensitivity to poor catheter contact can be improved in the future by changing the hard threshold of $\frac{\pi }{2}$ to a soft threshold, in which case the region splits will have a smooth curved boundary as opposed to the current straight-line boundary. We found only a small increase in the number of catheter placements (an increase from 6.5 to 8.8 average catheter placements) as the catheter-placement error was increased. This shows that the algorithm’s navigation regulates the number of iterations to be as small as possible, which is according to the nature of the underlying algorithmic approach that was used in ASAP [22].

In addition, we used three cases of clinical AF to indicate the potential of ASAP for detecting AF sources with repeating patterns. Our analysis was performed retrospectively on the collected electrogram signals. For each patient, an ASAP map was constructed that was overlaid on the patient’s left atrial anatomy. The generated maps indicated the presence of an AF source, which was mainly in the PV area. Given that the mapping was performed before the patients’ first PV ablation, this observation was expected and is also consistent with the prior FIRM mapping studies that report 40–50% of AF sources exist near PVs [28]. Another point to consider is that our study was performed using 2.5-s clinical electrograms. Analysis of longer duration of the data may reveal additional AF sources in the atria. Additional prospective clinical studies are required to investigate the effect of data duration on the location and number of the detected AF sources. The full potential of the algorithm in navigating the optimal placement of a catheter in the atria and successful localization of an AF source warrants further clinical investigation, where the algorithm is applied after PV ablation or redo-ablations.

### Comparison of ASAP with FRIM mapping algorithms

The existing clinical FIRM mapping utilizes a dedicated whole-atrial basket catheter to obtain global endocardial signals from an atrial chamber and then locates AF source origin using the phase mapping technique. We applied this method to our simulation cases. The FIRM mapping method resulted in an average successful detection rate of less than 50% in all the test cases except for the 3D simulations, where the average detection rate was in the 60% range. This is while our analysis of the ASAP algorithm showed a performance of greater than 95% in all the cases. Such a behavior indicates the capability of ASAP in identifying AF sources with complex activation patterns such as figure-of-eight, transmural, anatomical barrier, and chaotic reentries, where the clinical FRIM mapping is challenged. It is also worth mentioning that our ASAP mapping algorithm does not require the use of basket catheters, and it can be implemented using any high-resolution, conventionally used multipole catheters.

### Clinical significance, limitations, and future work

The ASAP algorithm can be integrated, as an add-on software, into the existing AF mapping systems used in clinical electrophysiology (EP) and offers various improvements to the existing approaches for localizing of AF sources. ASAP can be applied on the 3D atrial geometry of a patient obtained preoperatively using a 3D anatomic imaging system such as KODEX-EPD cardiac imaging and mapping system (Philips Healthcare, OH). It then can prospectively analyze electrogram recordings at every catheter placement to generate a spatial distribution of the probability of the presence of an AF source and also make recommendations for the treating clinician for subsequent catheter placements such that the algorithm can delineate the meandering path of an AF source. To the best of our knowledge, ASAP is the first AF mapping system that navigates a catheter in the atria to intelligently sample the preoperatively obtained atrial anatomic image of a patient without the need for time-consuming point-by-point mapping of the entire atria. Hence, in a clinical setting, ASAP mapping has the potential to locate AF sources faster (within a few catheter placements) than the existing electrogram-guided methods. Furthermore, as the catheter is placed on different locations in the endocardium, the algorithm provides visual feedback about the spatial probability map of the presence of a meandering AF source. This map can be used by the clinician to locate the ablation targets and holistically formulate ablation strategies. The ASAP algorithm does not require specialized hardware; it can be integrated with current electroanatomic mapping systems and can be used with any conventional high-resolution catheters (no whole-chamber basket catheters required). We validated the applicability of ASAP using both a Lasso and a spiral catheter. Given that the method utilizes the local activation time of the electrograms, the method can be extended to use a wide range of diagnostic catheters, such as Pentaray and Grid mapping catheter. Additionally, the method can detect any source type (i.e., reentry or focal) and does not have to make any presumptions about the type of AF source. ASAP mapping is designed to detect the area where the source is meandering. Such a characteristic opens discussion on how to create successful ablation lines when only an area of the source is known. This limitation can be addressed by developing customized ablation strategies such as a line-of-block lesion through the detected area connecting the closest boundary, similar to isthmus lines [26]. Our computer simulation and retrospective clinical studies indicated a significant potential of ASAP mapping in detecting repeating-pattern AF sources. Validation of ASAP in successful AF ablation warrants further prospective human investigation.

## Conclusions

In this paper, we developed a novel AF source mapping algorithm to probabilistically map the meandering AF source areas by iteratively navigating a 20-electrode single-loop or double-loop circular catheter that is commonly used in AF ablation procedures. We designed a novel algorithmic approach that uses the variations in the electrogram characteristics to guide the catheter placements towards a meandering repeating-pattern AF source. At every catheter placement, the algorithm estimates the direction of the wavefront and accordingly makes a recommendation about the subsequent placement of the catheter. As the catheter is moved across the endocardium, the algorithm constructs an ASAP map overlaid on the atrium anatomy informing the clinician about the areas with a high probability of the presence of a meandering AF source. Once some predefined criteria are met, the algorithm provides the region with the highest probability of the presence of the trajectory path of a meandering AF source. Our testbed included simulations of human AF with different types and number of AF sources in single and two-layer 2D atrial tissue and realistic 3D geometry. The algorithm was able to successfully map and delineate AF sources within less than eight catheter placements and with over 95% accuracy in all cases, which was significantly greater than the performance of the FIRM mapping technique (range: 29–66%). Our clinical proof-of-concept evaluation further demonstrated the feasibility of ASAP for detecting AF sources in clinical AF data. The developed computational AF mapping system has the potential for patient-customized analysis of the wave propagation patterns in human AF, thereby enabling the detection of novel ablation targets in patients undergoing AF ablation for a successful AF ablation therapy. Future investigations and clinical trials with a more significant number of clinical AF are needed to validate ASAP as a new mapping method.

## Methods

### Study design and procedures

This section describes the methods used for generating simulated and retrospective clinical human AF data.

#### Human AF simulations

A10 cm × 10 cm 2D atrial tissue with a spatial resolution of 0.25 mm (Fig. 1) was used for the 2D atrial model. For the 3D atrial model, we used a version of the Harrild and Henriquez real 3D human anatomy model [29] that consists of the left atrium, right atrium, Bachmann’s bundle, pectinate muscles, left atrial appendage, fossa ovalis, and the superior and inferior vena cava with the addition of the coronary sinus and inferior interatrial connection [30]. We simulated fibrosis in both 2D and 3D atrial models. In 2D, fibrosis was introduced by randomly removing electrical connections between neighboring myocytes oriented along horizontal fiber orientation, with an average septum length of 2.5 mm. The 3D fibrosis was created in the form of 2.5 × 2.5 × L mm blocks at 400 randomly sampled (uniformly distributed) locations, where L, the length of the fibrotic septum, was sampled from a Poisson distribution with an average length of 2.5 mm. Next, we used the Nygren et al. [31] human atrial cell model, which is based on average voltage-clamp data recorded from isolated single myocytes from typical bulk atrial tissue, to simulate human AF on 2D and 3D atrial tissue. The reentry sources were implemented with a cycle length of about 140 ms in the 2D case and 170 ms in the 3D case [32]. We also simulated a figure-of-eight reentry, which is common in human AF [8], by generating two closely spaced reentry cores (Fig. 1c). In the case of the two-layer 2D model, we initiated a reentry in the epicardial layer. An anatomical barrier in the form a myocardial patchy scar was created by randomly removing 50% of electrical connections within 2.25 cm^2^, which resulted in a functional macroreentry anchoring to the anatomical barrier (Fig. 1d). A stable fibrillary cluster of reentry activities was simulated by introducing spatial heterogeneity in the inward rectifier channel conductance by increasing the *I*_K1_ conductance, which has been shown to be linked to AF‐related remodeling [33], in the lower half of the simulated area. The trajectory path of the meandering reentry sources is shown by black tracings in Fig. 1. To generate a focal source, we used a pacing stimulus with an amplitude of 20 mV on an area of 1.25 mm × 1.25mm. This resulted in a focal source with a cycle length of about 150 ms in the 2D and 250 ms in the 3D simulations.

#### Catheter and electrogram simulations

**Fig. 6:**
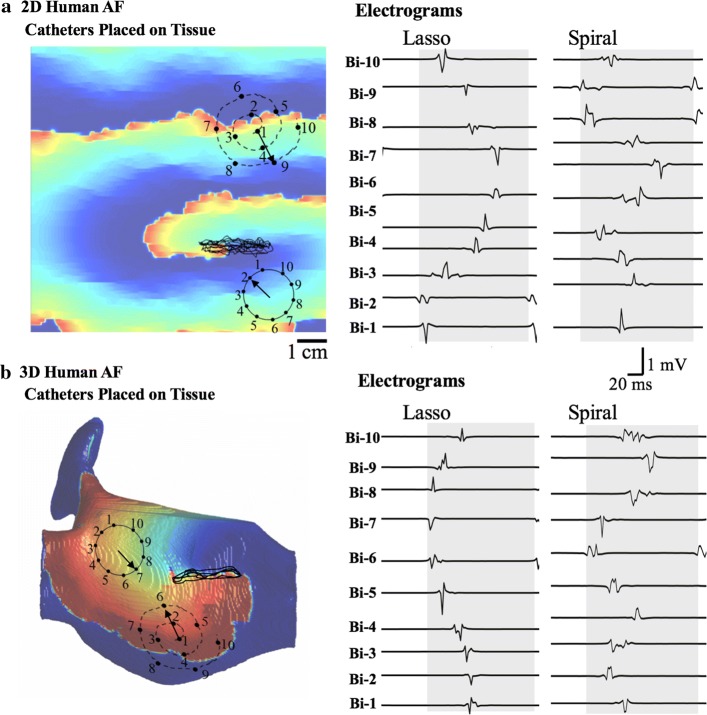
Electrograms of Lasso and spiral catheters in a **a** 2D simulation and **b** 3D simulation. The black arrows indicate the calculated principal wave direction (PWD). Each gray box indicates one detected activation cycle. The black tracings indicate the trajectory of the meandering source core

A 20-electrode Lasso circular catheter (Biosense Webster Inc., CA) with 15 mm diameter and 4.5-1-4.5 mm electrode spacing and a 20-electrode spiral catheter (Reflexion, St. Jude Medical Inc., MN) with 2-7-2 mm electrode spacing and an outer diameter of 25 mm with 20 voltage-sensing unipolar electrodes were simulated. Each of these unipolar electrodes was arranged in pairs of two, virtually forming a bipolar electrode. In the case of the 2D simulations, the electrodes’ coordinates were directly placed on the 2D tissue. For the simulations in the real 3D anatomy, we used our method [34] to project and register the plane of principal curvature of the catheter to the 3D surface. The unipolar electrode coordinates were used to acquire the transmembrane potentials from the corresponding location on the atrial tissue. The intracardiac electrograms were calculated from the transmembrane potentials [35] with a sampling interval of 2 ms. The bipolar electrograms were then calculated by subtracting the electrograms at the unipolar pairs and were used by the ASAP mapping algorithm. Examples of the simulated electrograms in 2D and in the 3D atrial anatomy using a Lasso and spiral catheter are shown in Fig. 6.

#### Transformation of 3D anatomy to 2D

For the transformation process, we unwrapped the 3D atrial structure into a 2D sheet using multi-dimensional scaling while minimizing the distance distortions between the vertices (nodes) in the reconstructed 3D model [36]. Additional file 4: Figure S2 illustrates the transformation steps as performed in our work. The average transformation error of a point was 3.80 ± 1.53 mm, which was calculated based on the average error in the 3D after transformation from 2D and then back to 3D.

#### Clinical AF data

We used clinical AF data collected retrospectively from three patients undergoing their first AF ablation, two with persistent AF (Subject 1 and Subject 2) and one with paroxysmal AF (Subject 3) to investigate the clinical applicability of ASAP. The study was approved by the institutional review board, and all patients provided written informed research consent. Transthoracic echocardiography and transesophageal echocardiography were performed 1 day before ablation in order to evaluate left ventricular ejection fraction and LA size. The clinical characteristics of these subjects are given in Table 2. After the interatrial septal puncture, a 20-pole variable curve circular catheter with 15–25 mm diameter, 1 mm inter-electrode distance, (Lasso, Biosense Webster Inc, CA) was placed in the left atrium through a guide sheath. At each recording location, 10 bipolar electrograms (30–500 Hz) were simultaneously recorded for 2.5 s at a sampling interval of 1 ms only after catheter stability was achieved. The details of the procedure are described by Ghoraani et al. [25].

**Table 2:** Patient characteristics

Age (year)	40–59	Body mass index (kg/m^2^)	28–31
Male (female)	3 (0)	Left ventricular ejection fraction (%)	56–68
Paroxysmal (persistent) AF	1 (2)	Left atrial volume (mm)	67–102
Hypertension	1	Antiarrhythmic drugs	2
Diabetes	0	Amiodarone	2

### AF source area probability (ASAP) mapping algorithm

**Fig. 7:**
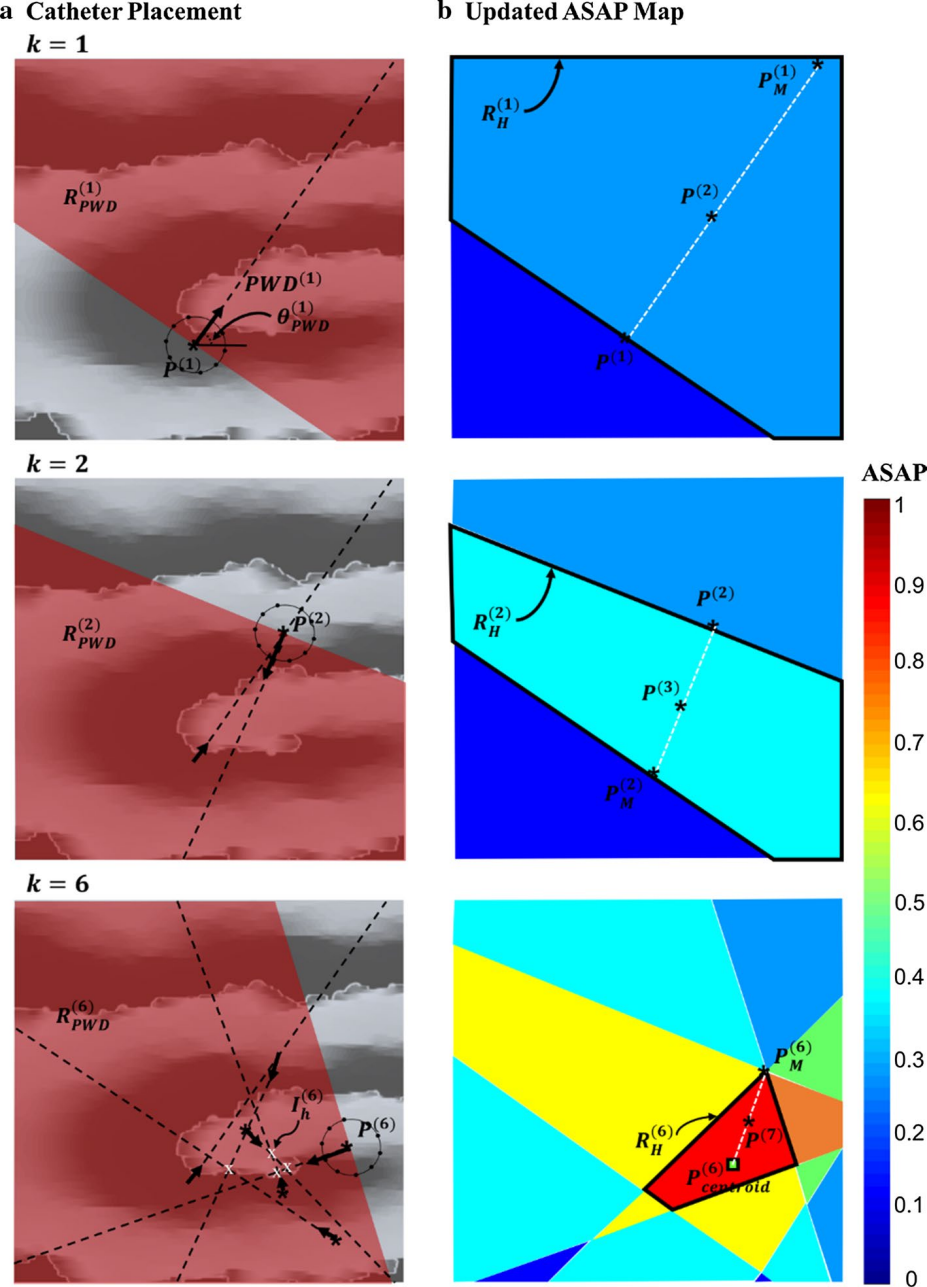
Catheter placements and generated AF source area probability (ASAP) map at catheter placements of *k *= 1, 2, and 6 in the simulation case of Fig. 1a. **a** The principal wave direction (PWD) vector is shown at every catheter placement. Priority map *α*^(*k*)^ is assigned to 1 at the region of PWD vector $R_{\text{PWD}}^{\left( k \right)}$ (the red shaded area). The intersections of all the PWD vectors are obtained as the number of catheter placement increases. **b** The ASAP map is updated using the priority map. The boundary coordinates $R_{H}^{\left( k \right)}$ of the ASAP area with the highest value is identified. The coordinate $P^{{\left( {k + 1} \right)}}$ of the next catheter placement is determined using either the PWD-based method (cases in *k* = 1, 2) or centroid-based method (*k* = 6 case)

The algorithm for the four phases in ASAP is outlined in Algorithm 1 and described in this section. Note that because of the 3D-to-2D transformation (Transformation of 3D anatomy to 2D of the “Methods” section), all the phases are the same in the 2D and 3D AF simulations. Figure 7 illustrates the catheter placement and ASAP map construction at three catheter placements of $k = 1,2$, and $k = 6$.
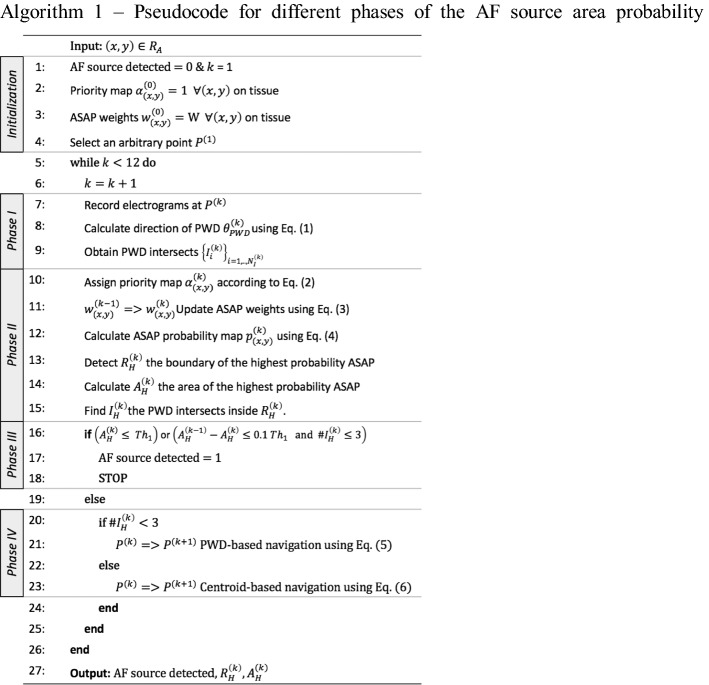

#### Phase I—electrogram processing

At every placement of the catheter, 1 s (in simulation AF) and 2.5 s (in clinical AF) of 10 bipole electrograms are collected and processed using the following steps:

- The local activation times of each electrogram are detected as the time of the maximum negative deflection within a 50-ms duration. Cycle length is calculated as the median value of the time delays between any two subsequent local activations in every electrogram.
- A customized program [37] was developed to identify the local activation times associated with the same wavefront as one cycle. The gray boxes in Fig. 6 indicate the detected cycles.
- PWD estimates the direction opposite the direction of the propagating AF source wavefront relative to the current location of the catheter. The angle of the PWD vector, $\theta_{\text{PWD}}^{\left( k \right)}$, at the $k$ th catheter placement is calculated using Eq. (1). ${\text{FAB}}_{c}$ is the coordinate of the first activated bipole for cycle $c$, where $c$ varies from 1 to $N$, the number of cycles detected in 1 s of the recording. $P$ is the coordinates of the catheter center and $d_{c} e^{{j\theta_{c} }}$ represents the polar coordinates of a vector that connects the catheter center to the earliest activated bipole. The arrows in Figs. 6 and 7a illustrate the calculated PWD vector for the displayed catheter placements.
  $$d_{c} e^{{j\theta_{c} }} = {\text{FAB}}_{c} - P^{\left( k \right)}$$
  $$c \in \left[ {1, \ldots ,N} \right]$$
  1 $$\theta_{\text{PWD}}^{\left( k \right)} = \frac{{\mathop \sum \nolimits_{c = 1}^{N} \theta_{c} }}{N}$$
- The coordinates of all the intersections of the PWD vectors from the first catheter placement until the current one are determined as $\left\{ {I_{i}^{\left( k \right)} } \right\}_{{i = 1,.., N_{I}^{\left( k \right)} }}$. For this purpose, from every catheter placement $k$, a line is extended in the direction of the corresponding PWD vector. Once all the $N_{I}^{\left( k \right)}$ intersections are determined, the algorithm provides their coordinates to the next phases.

#### Phase II—ASAP map update

There are two steps involved in this phase. Step 1 provides the ASAP map, which represents a spatial probability of the presence of an AF source on the atrial anatomy by assigning probability values $p_{{\left( {x,y} \right)}}^{\left( k \right)}$ to every $\left( {x,y} \right) \in R_{A}$ coordinates on the anatomy which can be obtained from pre-operative imaging as an alternative to the electroanatomic map. Step 2 characterizes the generated ASAP map. It identifies the largest area with the highest ASAP value and provides this area’s boundary coordinates $\left( {x_{h} ,y_{h} } \right) \in R_{H}^{\left( k \right)}$ and its size $A_{H}^{\left( k \right)}$ as well as the coordinates of the PWD intersections $I_{H}^{\left( k \right)}$ that fall inside this area (Fig. 7). These values are used in Phase III to identify whether an AF source area is delineated and in Phase IV to provide the coordinates of the next catheter placement.

- Step 1 constructs the ASAP probability map based on a weight value $w_{{\left( {x,y} \right)}}^{\left( k \right)}$ that is assigned to every coordinate. Before the algorithm starts, the weight values $w_{{\left( {x,y} \right)}}^{\left( 0 \right)}$ are initialized to a constant value $W$. Once a catheter is placed, the weight values are updated according to $\theta_{\text{PWD}}^{\left( k \right)}$, the angle of the PWD vector, in two steps. First, a priority value $\alpha_{{\left( {x,y} \right)}}^{\left( k \right)}$ is assigned to every coordinate. As shown in Eq. (2), the area $R_{PWD}$ that falls in $\pm \frac{\pi }{2}$ degrees from the direction of the PWD vector is assigned to one indicating that there is a higher chance of the presence of an AF source in that direction. The priority values are initialized to $\alpha_{{\left( {x,y} \right)}}^{\left( 0 \right)} = 1,$ assuming that the chance of the presence of the core of an AF source is equivalent everywhere on the tissue. Second, the weight values $w_{{\left( {x,y} \right)}}^{\left( k \right)}$ are updated by comparing the priority values at the current $\alpha_{{\left( {x,y} \right)}}^{\left( k \right)}$ and previous $\alpha_{{\left( {x,y} \right)}}^{{\left( {k - 1} \right)}}$ catheter placement as shown in Eq. (3). The weight values are updated such that the weights at the coordinates that were consistently in a high priority area increase by $\delta$, or decrease by $\delta$ if they are consistently at a low priority area. If the priority values of the current and previous catheter placements are different, the algorithm retains the weight values in order to reduce the selectivity of the algorithm to any significant errors in the estimation of the PWD vector. The value of constant $\delta$ is selected such that the weight values do not become negative until $K =$ 12 catheter placements occurred: $\delta = \frac{W}{K - 1}$ with *W* = 500. Note that the values of $W$ and $K$ are arbitrarily selected; however, the algorithm’s performance will not be affected as long as the relationship above between $\delta , W$, and $K$ holds. Once the ASAP weights are updated, the ASAP probability map is calculated based on the weight values $w_{{\left( {x,y} \right)}}^{\left( k \right)}$ using a normalizing procedure shown in Eq. (4):
  2 $$\alpha_{{\left( {x,y} \right)}}^{\left( k \right)} = \left\{ {\begin{array}{ll} 1 & \quad {{\text{if}}\;\left( {x,y} \right) \in R_{\text{PWD}} } \\ 0 & \quad {\text{Otherwise}} \\ \end{array} } \right.$$
  3 $$w_{{\left( {x,y} \right)}}^{{\left( k \right)}} = \left\{ {\begin{array}{*{20}l} {w_{{\left( {x,y} \right)}}^{{\left( {k - 1} \right)}} + \delta } \hfill & {{\text{if }}\alpha _{{\left( {x,y} \right)}}^{{\left( k \right)}} = \,1\,\,\& \,\,\alpha _{{\left( {x,y} \right)}}^{{\left( {k - 1} \right)}} = 1} \hfill \\ {w_{{\left( {x,y} \right)}}^{{\left( {k - 1} \right)}} - \delta } \hfill & {{\text{if}}\,\alpha _{{\left( {x,y} \right)}}^{{\left( k \right)}} = 0\,\,\& \,\,\alpha _{{\left( {x,y} \right)}}^{{\left( {k - 1} \right)}} = 0} \hfill \\ {w_{{\left( {x,y} \right)}}^{{\left( {k - 1} \right)}} } \hfill & {{\text{Otherwise}}} \hfill \\ \end{array} } \right.$$
  4 $$p_{{\left( {x,y} \right)}}^{\left( k \right)} = \frac{{w_{{\left( {x,y} \right)}}^{\left( k \right)} }}{{\mathop \sum \nolimits_{{\left( {x,y} \right) \in R_{A} }} w_{{\left( {x,y} \right)}}^{\left( k \right)} }}$$
- Step 2 characterizes the updated ASAP map to detect the largest area with the highest ASAP value and provides this area’s border coordinates $\left( {x_{h} ,y_{h} } \right) \in R_{H}$ and its size $A_{H}^{\left( k \right)}$. First, the algorithm identifies the coordinates of the boundaries from the spatial derivative of the ASAP map by obtaining the coordinates with a non-zero derivative value. Next, the boundary coordinates of the areas with the highest ASAP value are identified. To make this happen, we select the non-zero derivative coordinates at which one of the four neighboring coordinates holds the highest ASAP value. This process provides all the boundaries of all the areas with highest ASAP value. Note that there may be more than one such area, so the algorithm continues by detecting all of those distinct areas and identifying the one with the largest area size. In order to find all the existing distinct areas, we converted the ASAP map into a binary image where the coordinates of the boundaries of all the areas with highest ASAP value are set to one and the rest to zero. Then, we used a Flood-fill algorithm [38] to assign each area to its corresponding boundary coordinates. Flood-fill algorithms are commonly used in imaging applications to detect areas enclosed by a closed boundary starting from a random seed. Here, we use a Flood-fill algorithm by starting the seed at the known highest ASAP values and finding the distinct areas. Once all the distinct areas with the highest ASAP value are identified, the algorithm calculates a numerical area by counting the number of coordinates inside of each distinct area and selects the area with the largest area size. The boundary coordinates of this selected area is denoted as $\left( {x_{h} ,y_{h} } \right) \in R_{H}^{\left( k \right)}$, the numerical sized of this area $A_{H}^{\left( k \right)}$, and the PWD intersects inside this area $I_{H}^{\left( k \right)}$ are used in the next phases.

#### Phase III—AF source delineation criteria

The algorithm stops its search once one of the following two source delineation criteria is met or the number of catheter placements exceeds 12. The first criterion is met when the algorithm narrows down the ASAP area with the highest probability to a small region, meaning that the algorithm delineated the trajectory path of a meandering AF source or part of its trajectory inside the highest probability ASAP area. This criterion is quantified as $A_{H}^{\left( k \right)} \le {\text{Th}}_{1}$, where $A_{H}^{\left( k \right)}$ indicates the size of the area of the current ASAP map that has the highest probability value, and ${\text{Th}}_{1} = 2\pi r^{2}$ is twice of the area of a Lasso catheter, where $r$ is the catheter radius $7.5\;\;{\text{mm}}$. The second criterion is met when there is a small change in the area of the highest probability ASAP area as the catheter is navigated from $k - 1$ (past) to $k$ (current) catheter placement, but there are at least three PWD intersections inside this area. The reason for this criterion is that once the catheter is placed a certain distance from the core of the source, the catheter navigation could be small, which results in a small update to the mask. However, the PWD vectors are expected to point towards the core of the AF source. Hence, the presence of enough PWD intersections in the highest probability ASAP area could indicate the presence of the core of a meandering AF source in the area. We quantified this criterion by $A_{H}^{{\left( {k - 1} \right)}} - A_{H}^{\left( k \right)} \le 0.1 {\text{Th}}_{1}$, and the presence of at least three PWD intersects $I_{H}^{\left( k \right)}$ inside the area of $\left( {x_{h} ,y_{h} } \right) \in R_{H}^{\left( k \right)}$.

#### Phase IV—catheter navigation

The algorithm makes a recommendation for the location of the next catheter placement using the angle of the PWD vector and the ASAP map at the current catheter placement ($k$). Depending on $I_{H}^{\left( k \right)}$, the number of the PWD intersections in the highest probability ASAP area, two types of catheter guidance are performed:

- PWD-based navigation moves the next placement of the catheter $P^{{\left( {k + 1} \right)}}$ to half the distance from the current catheter placement $P^{\left( k \right)}$ to the boundary of the highest probability ASAP area $\left( {x_{h} ,y_{h} } \right) \in R_{H}$ in the direction of the PWD vector with an angle of $\theta_{\text{PWD}}^{\left( k \right)}$. This type of navigation is initiated when the number of coordinates in $I_{H}^{\left( k \right)}$ is less than 3. The mathematical formulation is described in Eq. (5) and illustrated in Fig. 7 ($k = 1$ and $2$). The parameter $d_{h} e^{{j\theta_{h} }}$ indicates the polar form of the distance from the center of the current catheter to the boundary of the highest probability ASAP area. The index $M$ indicates the boundary coordinates $P_{M}^{\left( k \right)}$ of this area that are in the same angle as the PWD vector. Note that there could be more than one such boundary coordinate. We select the one that is the farthest (i.e., with maximum $d_{M}$). As shown in Eq. (5), the algorithm recommends the next catheter placement at $\frac{{d_{M} }}{2}e^{{j\theta_{M} }}$, which is the midpoint between the current location of the catheter and boundary coordinate $M$:
  $$d_{h} e^{{j\theta_{h} }} = P^{\left( k \right)} - p_{{\left( {x_{h} ,y_{h} } \right)}}$$
  $$M = {\text{arg min}}\left| {\theta_{h} - \theta_{PWD}^{\left( k \right)} } \right| \left( {x_{h} ,y_{h} } \right) \in R_{H}$$
  5 $$P^{{\left( {k + 1} \right)}} = \frac{{d_{M} }}{2}e^{{j\theta_{M} }}$$
- Centroid-based navigation is initiated when there are at least three $I_{H}^{\left( k \right)}$ PWD intersections in the highest probability ASAP area. The idea behind this type of navigation is that the region with the PWD intersections most likely is close to the AF source. Hence, the algorithm places the catheter in the highest probability ASAP area such that it can reduce the size of the highest probability ASAP area in the next catheter placement. As shown in Fig. 7 ($k = 6$) and Eq. (6), the next catheter placement will be at the midpoint of the centroid $P_{\text{centroid}}^{\left( k \right)}$ of the intersections $I_{H}^{\left( k \right)}$ to $P_{M}^{\left( k \right)}$, the farthest points on the boundary of the highest probability ASAP area $R_{H}^{\left( k \right)} : d_{h} e^{{j\theta_{h} }} = P_{\text{centroid}}^{\left( k \right)} - p_{{\left( {x_{h} ,y_{h} } \right)}}$
  $$M = {\text{arg max}}\left| {d_{h} } \right| \left( {x_{h} ,y_{h} } \right) \in R_{H}$$
  6 $$P^{{\left( {k + 1} \right)}} = \frac{{d_{M} }}{2}e^{{j\theta_{M} }}$$

## Supplementary information

**Additional file 1: Movie S1.** The Transmembrane voltage map of figure-of-eight reentry (Fig. 1c).

**Additional file 2: Movie S2.** The Transmembrane voltage map of two-layer model (Fig. 1d).

**Additional file 3: Movie S3.** The Transmembrane voltage map of stable fibrillatory cluster of rotors (Fig. 1f).

**Additional file 4: Figure S1.** A few samples of fractionated electrograms generated in this study. (A) Simulation in Fig. 1a. (B) Simulation in Fig. 1f. (C) Simulation in Fig. 1e. We modeled tissue fibrosis and generated realistic fractionated electrograms by introducing collagenous septa according to a Poisson distribution with an average septa length of 2.5 mm and disrupted lateral coupling in 20% of the tissue. **Figure S2.** Transformation of 3D anatomy to 2D tissue. (A) Posterior view: The black dotted lines indicate the locations of the cut that was performed in MeshLab. (B) Lateral view: The gray arrows indicate the unwrapping direction. (C) 2D view: The transformed 2D equivalent with optimal inter-vertex distance deformation. LAA: left atrial appendage. LPVs: left pulmonary veins. RPVs: right pulmonary veins. **Figure S3.** (A) The catheter placement and the AF source area probability (ASAP) map in the simulation case of Fig. 1e, and (B) in its corresponding transformed 2D model. When evaluating the algorithm in the 3D model, the catheter was placed and the electrograms were obtained in the 3D domain, but the algorithm was performed in the 2D domain. The average spatial error between the two domains was 2.8 mm. **Figure S4.** Repeating-pattern rotor source (cycle length ≈ 166 ms) is recorded at catheter placement L1 in subject 1. The gray box indicates a wave propagation cycle detected by our customized program [37], which assigns the local activation times associated with the same wavefront as one cycle. The rotor source is identified as a sequential temporal local activation around the circular catheter such that it spans through one cycle length.

## Data Availability

The datasets and materials are available from the corresponding author upon request.

## Abbreviations

AF: Atrial fibrillation
PVs: Pulmonary veins
ASAP: AF source area probability
PWD: Principal wave direction
2D: 2-Dimensional
3D: 3-Dimensional
