# Supplementary material for: Atrial fibrillation source area probability mapping using electrogram patterns of multipole catheters
Source: Biomed Eng Online. 2020 May 5;19:27. doi: 10.1186/s12938-020-00769-0 (PMC7201756; doi:10.1186/s12938-020-00769-0)

Figure S1 - A few samples of fractionated electrograms generated in this study. (A) Simulation in Figure 1A. (B) Simulation in Figure 1F. (C) Simulation in Figure 1E. We modeled tissue fibrosis and generated realistic fractionated electrograms by introducing collagenous septa according to a Poisson distribution with an average septa length of 2.5mm and disrupted lateral coupling in 20% of the tissue.


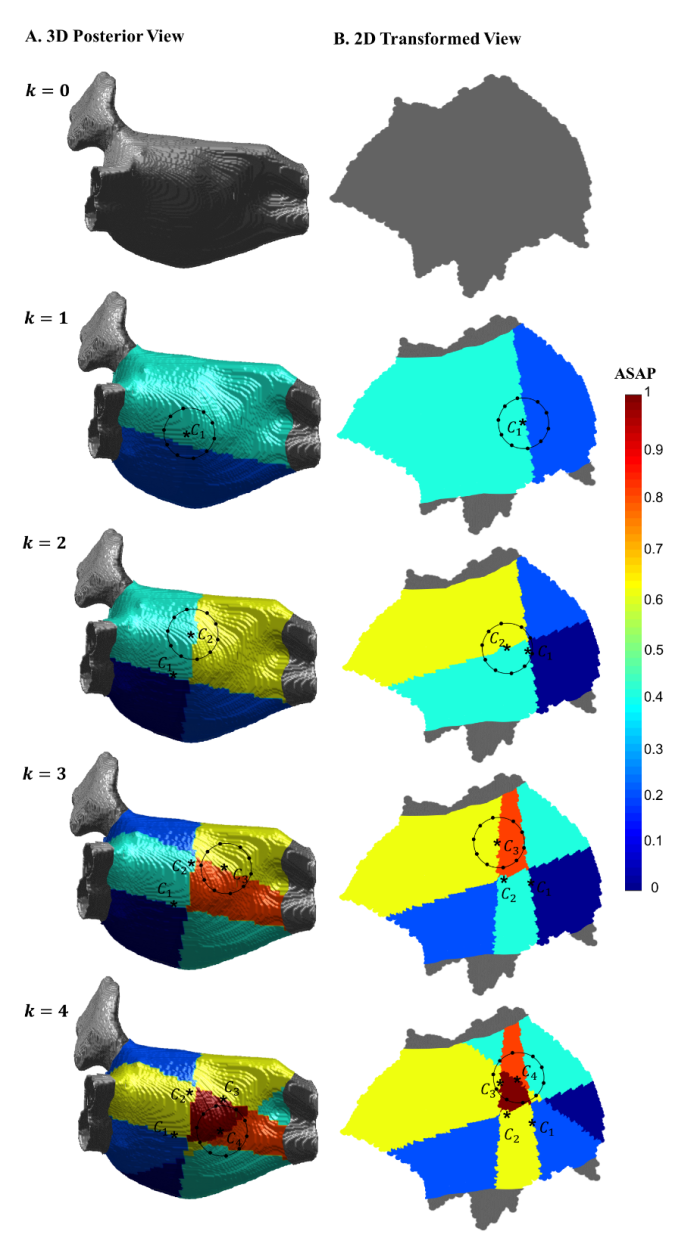

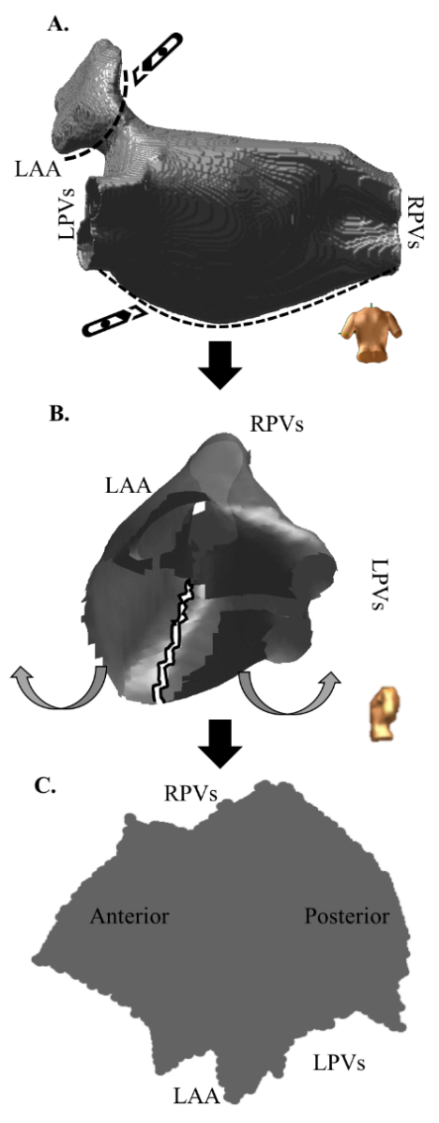


Figure S2 - Transformation of 3D anatomy to 2D tissue. (A) Posterior view: The black dotted lines indicate the locations of the cut that was performed in MeshLab. (B) Lateral view: The gray arrows indicate the unwrapping direction. (C) 2D view: The transformed 2D equivalent with optimal inter-vertex distance deformation. LAA: left atrial appendage. LPVs: left pulmonary veins. RPVs: right pulmonary veins.

Figure S3 – (A) The catheter placement and the AF source area probability (ASAP) map in the simulation case of Figure 1E, and (B) in its corresponding transformed 2D model. When evaluating the algorithm in the 3D model, the catheter was placed and the electrograms were obtained in the 3D domain, but the algorithm was performed in the 2D domain. The average spatial error between the two domains was 2.8mm.

Figure S4 - Repeating-pattern rotor source (cycle length $\approx$ 166 ms) is recorded at catheter placement L1 in subject 1. The gray box indicates a wave propagation cycle detected by our customized program [37], which assigns the local activation times associated with the same wavefront as one cycle. The rotor source is identified as a sequential temporal local activation around the circular catheter such that it spans through one cycle length.


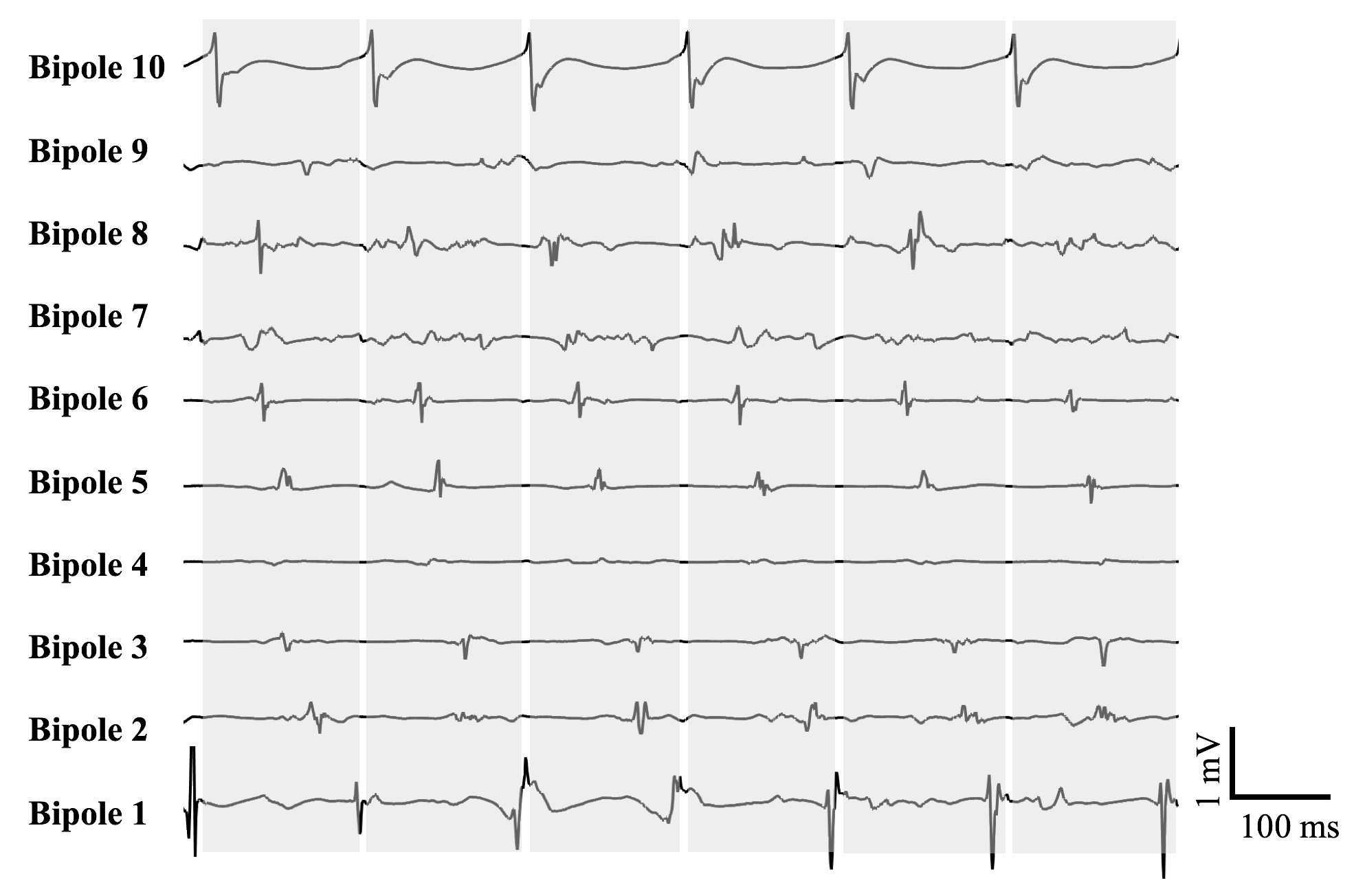

Supplement: Supplementary file 4 — Additional file 4: Figure S1. A few samples of fractionated electrograms generated in this study. (A) Simulation in Fig. 1a. (B) Simulation in Fig. 1f. (C) Simulation in Fig. 1e. We modeled tissue fibrosis and generated realistic fractionated electrograms by introducing collagenous septa according to a Poisson distribution with an average septa length of 2.5 mm and disrupted lateral coupling in 20% of the tissue. Figure S2. Transformation of 3D anatomy to 2D tissue. (A) Posterior view: The black dotted lines indicate the locations of the cut that was performed in MeshLab. (B) Lateral view: The gray arrows indicate the unwrapping direction. (C) 2D view: The transformed 2D equivalent with optimal inter-vertex distance deformation. LAA: left atrial appendage. LPVs: left pulmonary veins. RPVs: right pulmonary veins. Figure S3. (A) The catheter placement and the AF source area probability (ASAP) map in the simulation case of Fig. 1e, and (B) in its corresponding transformed 2D model. When evaluating the algorithm in the 3D model, the catheter was placed and the electrograms were obtained in the 3D domain, but the algorithm was performed in the 2D domain. The average spatial error between the two domains was 2.8 mm. Figure S4. Repeating-pattern rotor source (cycle length ≈ 166 ms) is recorded at catheter placement L1 in subject 1. The gray box indicates a wave propagation cycle detected by our customized program [37], which assigns the local activation times associated with the same wavefront as one cycle. The rotor source is identified as a sequential temporal local activation around the circular catheter such that it spans through one cycle length. [file 12938_2020_769_MOESM4_ESM.docx]
